# Marginal bone level changes and soft-tissue esthetics around implants with convergent transmucosal profiles: 10-year retrospective analysis

**DOI:** 10.1007/s00784-026-07207-w

**Published:** 2026-10-12

**Authors:** Nicola Alberto Valente, Gabriele Caruso, Nicola Carrus, Davide Cauli, Alessandro Cattaneo, Hani Abd-Ul-Salam

**Affiliations:** 1 Division of Periodontology, School of Dental Medicine, Department of Surgical Sciences, Faculty of Medicine and Surgery, University of Cagliari, Cagliari, Italy; 2 College of Dentistry, American University of Iraq, Baghdad, Iraq; 3 Private Practice, Cagliari, Italy; 4 Faculty of Dental Medicine and Oral Health Sciences, McGill University, Montreal, Canada; 5 Faculty of Dentistry, University of Toronto, Toronto, Canada

**Keywords:** Dental implants, Convergent transmucosal profile, Marginal bone level, Pink esthetic score, Soft tissue stability

## Abstract

**Objectives:**

The aim of this retrospective study was to analyze the long-term stability of marginal bone levels (MBL) and the Pink Esthetic Score (PES) in a cohort of patients treated with tissue-level implants featuring a convergent transmucosal profile.

**Materials and methods:**

A retrospective longitudinal analysis was conducted on 108 patients who received 193 implants with a specific convergent neck design and were followed for over 10 years. MBL was measured on digital periapical radiographs at baseline (loading), short-term (1–2 years), medium-term (3–6 years), and long-term (7–10 years) intervals. Esthetic outcomes were evaluated using the PES at the same time points comparing clinical photographs with reference teeth. Data were analyzed using repeated measures analysis of variance.

**Results:**

MBL remained stable throughout the observation period, with mean values of 1.17 mm at short-term, 1.17 mm at medium-term, and 1.23 mm at long-term follow-up, showing no statistically significant changes over time (*p* = 0.454). Conversely, PES values demonstrated a statistically significant increasing trend (*p* < 0.001), improving from a baseline mean of 7.99 to 10.34 at the long-term evaluation. Significant improvements were noted between baseline and all subsequent time points.

**Conclusions:**

Within the limits of this retrospective study, tissue-level implants with a convergent transmucosal profile demonstrated stable marginal bone stability and a progressive improvement in soft tissue esthetics over a 10-year period. This specific convergent transmucosal geometry appears to favor positive peri-implant tissue adaptation and long-term stability.

## Introduction

In the field of modern dental implantology, the long-term success and stability of implants depend not only on osseointegration, which is the direct contact between bone and implant, but also on the establishment and maintenance of healthy peri-implant soft tissues. (Glauser et al., [13]; G. Caton et al., [7]. It has been increasingly recognized that the interaction between implants and the peri-implant soft tissues is of primary importance for the health and longevity of rehabilitations.Mattheos et al., [20], Valente, [33] The health of peri-implant tissues is crucial for long-term implant success.

A fundamental concept in this context is that of “supracrestal tissue attachment” (once known as “biological width”), a physiologically formed and stable dimension of soft tissues surrounding both natural teeth and implants.Caton et al., [7], Monje, [21], Rungtanakiat, [30] This barrier acts as a protective seal, preventing the entry of bacteria and other harmful substances and safeguarding the underlying osseointegration.Cochran et al., [10], Hermann, [15] Studies have shown that, if the minimum thickness of peri-implant tissues is not respected (e.g., if the crestal mucosa is thin, ≤ 2 mm), bone resorption consistently occurs. (Berglundh and Lindhe, [3]; Linkevicius et al. [17]; Gianfilippo et al. [12]) This implies that a certain minimum thickness of the peri-implant mucosa may be required, and that bone resorption may take place to allow a stable soft tissue attachment to form.Berglundh and Lindhe, [3]

The transmucosal profile of the implant, which is the portion that passes through the soft tissues, and thus influence the supracrestal tissue attachment, plays a crucial role in both esthetical and biological peri-implant features.González-Martín et al., [14], Mattheos, [20], Perez, [24] The design of this profile shapes the architecture of peri-implant tissues and contributes to maintaining their stability, significantly influencing the gingival margin level, papilla height, gingival architecture, and overall aesthetics. In bone level implants, the implant-abutment interface, or “microgap,” located at the most apical level of the transmucosal area, can serve as a reservoir for bacterial colonization and inflammatory infiltration, contributing to marginal bone loss.Abrahamsson et al., [1], Broggini, [4], Broggini, [5], Do Nascimento, [23] The concept of “platform switching” has been introduced to shift this microgap horizontally, away from the bone crest, favoring a significant reduction in MBL but also promoting the organization of collagen fibers in a circular arrangement around the implant, forming a kind of “annular seal” believed to stabilize soft tissues and protect the underlying bone from resorption.Chang et al., [8], Ciurana, [9], Enkling, [11], Maeda, [19], Rodríguez, [26]

In recent years, there has been an introduction of new convergent transmucosal profiles in dental implants, contrasting with traditional divergent or parallel profiles.Agustín-Panadero et al., [2], Canullo, [6], Morón-Conejo, [22], Valente, [33] These convergent designs combine the advantages of eliminating the microgap, being the implant tissue level, with those of platform switching, favoring a shift of the collagen fibers towards the center and coronal direction.Ciurana et al., [9] It is also suggested that convergent profiles can facilitate a more ideal positioning of the restoration margin and condition soft tissues.Ciurana et al., [9], Rodriguez, [27] The concept of a convergent transmucosal profile was introduced with the aim of reducing the bulk of the implant neck near the soft tissues, and one of the first profiles of this type likely proposed on the market is that of that of the implant system used in this study.

Despite promising short-term results, a significant gap remains in the literature regarding the long-term stability of these new convergent transmucosal designs. The present retrospective study aimed to analyze the long-term stability of hard and soft tissues in a cohort of patients treated with implants featuring a convergent transmucosal profile. The study intended to identify correlations between aesthetic parameters and bone stability, also evaluating the clinical implications of this particular implant design.

## Materials and methods

**Fig. 1:**
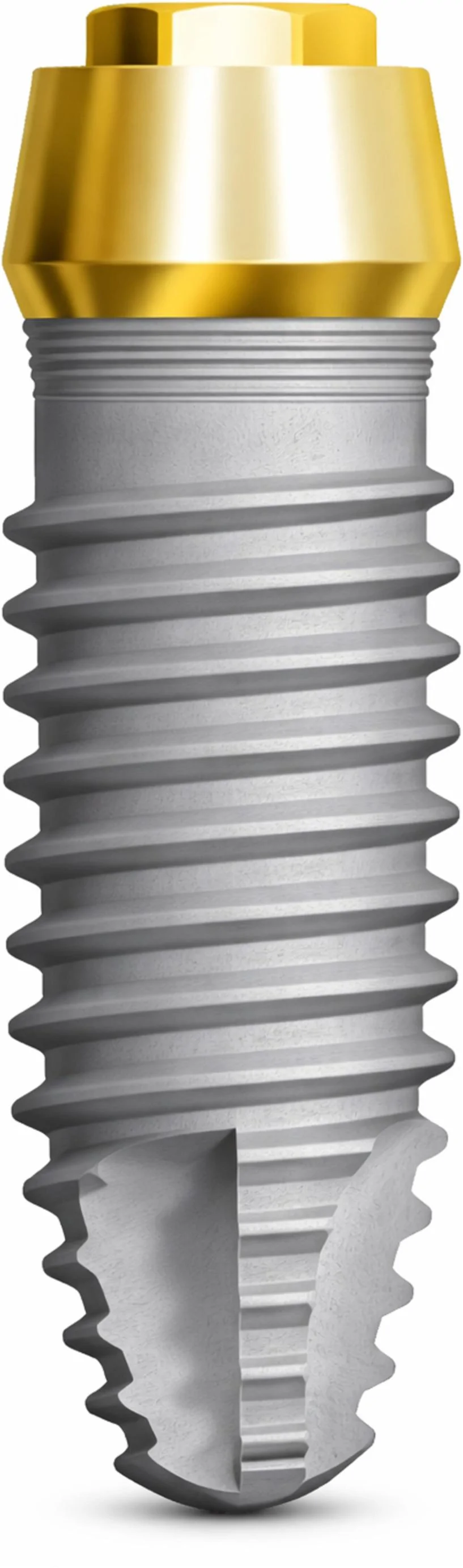
Vertical Neck Biotype implant design, with convergent transmucosal profile

The present study was designed as a retrospective longitudinal clinical analysis of a cohort of patients rehabilitated with dental implants featuring a convergent transmucosal profile (Vertical Neck, Biotype implant system, DEI Italia Srl, Mercallo VA, Italy) (Fig. 1). The study was conducted in accordance with the ethical principles established by the Declaration of Helsinki for medical research involving human subjects and was approved by the Regional Ethics Committee of the Autonomous Region of Sardinia (Prot. 8091, 19/03/2025). The study was registered at ClinicalTrials.gov (NCT07089407). This report follows the STROBE (Strengthening the Reporting of Observational Studies in Epidemiology) guidelines.(Von Elm et al. 2007) Data were collected from clinical records and the radiographic database of all treated patients who were followed for a follow-up period extending beyond 10 years. All sensitive information was anonymized to ensure participant privacy. A convenience sample was used, including all eligible patients/implants with available complete documentation and ≥ 10-year follow-up in the database.

### Study population and selection criteria

The study population consisted of patients who received fixed implant-supported rehabilitations. Participant selection was carried out by applying the following inclusion and exclusion criteria:

#### Inclusion criteria

- Patients aged ≥ 18 years at the time of implant placement.
- Presence of at least one implant with a convergent transmucosal neck design.
- Availability of a complete and readable periapical radiographic series, including: a radiograph taken at the time of implant insertion (T0), and at least one radiograph at follow-up intervals of 1–2 years, 3–6 years, and 7–10 years.
- Availability of intraoral clinical photographic documentation (buccal views) taken at the time of definitive prosthetic loading and at follow-up visits for esthetic evaluation.

#### Exclusion criteria

- Patients with uncontrolled systemic diseases (e.g., uncompensated diabetes mellitus, severe autoimmune diseases) that could affect bone metabolism or soft tissue healing.
- Patients classified as heavy smokers (> 10 cigarettes per day).
- Implant sites with incomplete or non-diagnostic clinical photographic or radiographic documentation.

### Implant characteristics

**Fig. 2:**
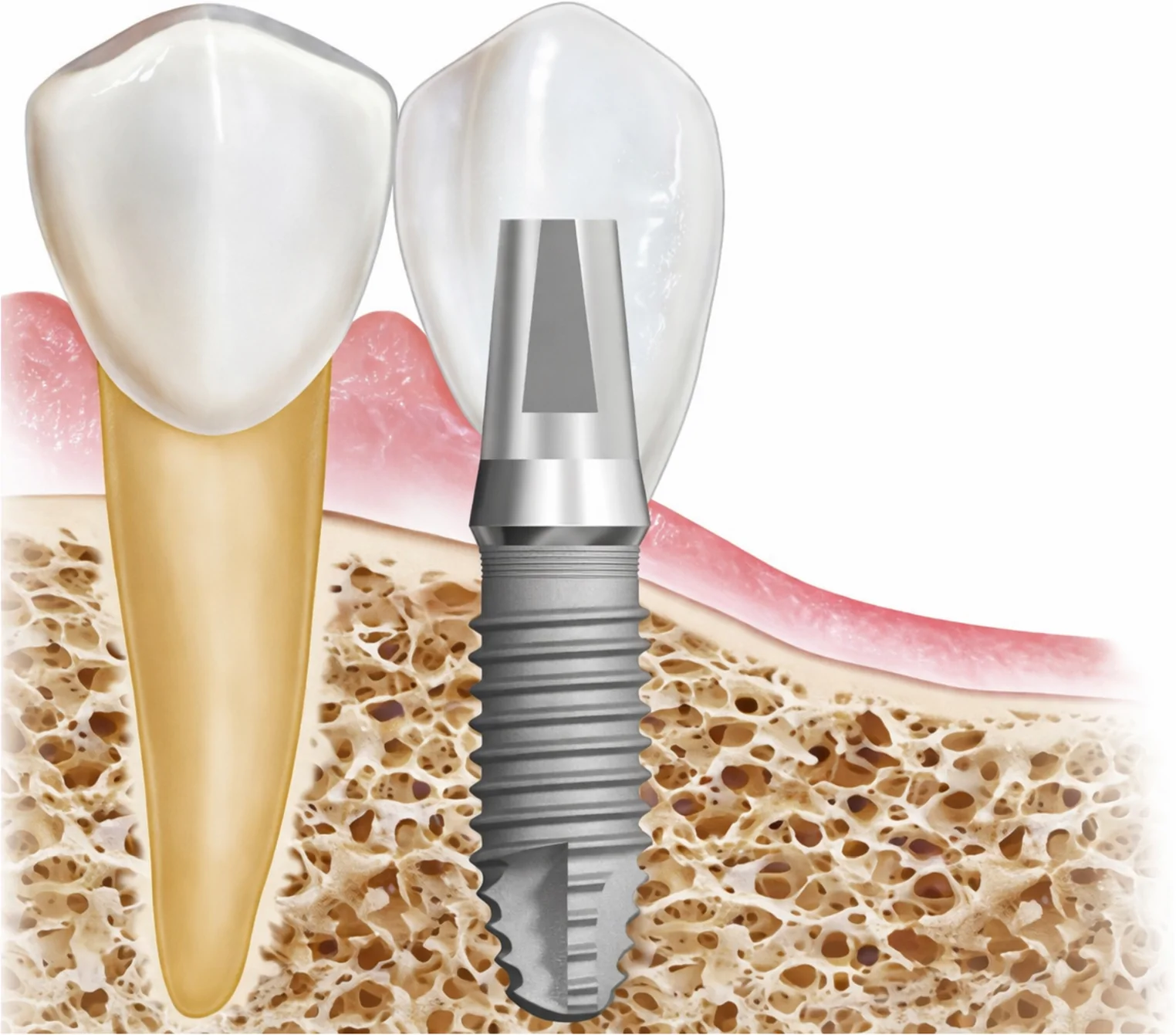
Schematic illustration of the prosthetic flexibility provided by the transmucosal implant design. The crown margin can be positioned at different vertical levels along the transmucosal implant surface or more coronally on the prosthetic component according to clinical requirements, and its position may be differentially modulated around the circumference of the same implant. The illustrated margin position represents one possible configuration and is not intended to indicate a standardized or mandatory position

The implants analyzed in this study feature a specific neck morphology characterized by a convergent transmucosal profile with a straight surface. This implant design was developed by Gabriele Caruso and Alessandro Cattaneo in 2010, with the European patent filed in 2011 (EP2770944, A1: 28.10.2011, B1: 13.06.2014, WIPO: 10CO2011A000051, IPC: A61C8/00). Consequently, it boasts extensive clinical usage that predates many similar transmucosal profile designs subsequently introduced to the market. This specific macro-geometry is intended to reduce the bulk of the implant neck at the crestal level and to promote coronal migration of collagen fibers, aiming to create a biological seal for marginal bone preservation and soft tissue support. The transmucosal profile of this implant was inspired by the vertical preparation technique of the prosthetic abutment, also known as the “finish line–free” technique. This implant zone is referred to as the “pre-abutment”. The prosthetic margin is not constrained to a predefined vertical position and may be placed at different levels along the transmucosal implant surface or more coronally on the prosthetic component, according to the clinical and prosthetic requirements. Moreover, the margin position may be differentially modulated around the circumference of the same implant. (Fig. 2).

### Data collection and assessment of clinical parameters

#### Radiographic analysis of marginal bone level (MBL)

Hard tissue stability was assessed by measuring the Marginal Bone Level (MBL) on digital periapical radiographs.

- Measurement method: Radiographic images were analyzed using open-source image processing software (ImageJ, National Institutes of Health, USA).
- Calibration: To correct for possible radiographic magnification or distortion, the software was calibrated using the known implant length or the known diameter of the implant platform as a reference.
- Reference points: MBL was calculated by measuring the vertical linear distance between the inferior margin of the pre-abutment (fixed reference) and the first visible point of bone-to-implant contact on both the mesial and distal aspects. Mesial and distal values were averaged to obtain a single MBL value per implant at each time interval.

#### Esthetic evaluation (PES)

The esthetic quality of peri-implant soft tissues was evaluated using the Pink Esthetic Score (PES), according to the method described by Fürhauser et al.(Fürhauser et al. 2005) The assessment was performed by comparing clinical photographs of the implant site with adjacent or contralateral reference teeth. The PES evaluates seven soft tissue variables, each scored 0, 1, or 2 (where 0 indicates the poorest outcome and 2 the best), for a maximum total score of 14. The seven variables assessed were:

1. Presence of the mesial papilla.
2. Presence of the distal papilla.
3. Level of the soft tissue margin (relative to the reference tooth).
4. Soft tissue contour (harmony of the gingival parabola).
5. Alveolar process deficiency.
6. Soft tissue color (including signs of inflammation or discoloration).
7. Soft tissue texture.

#### Examiner calibration

To ensure the reliability of radiographic and esthetic measurements, evaluations were performed by an experienced blind examiner who was not directly involved in the surgical or prosthetic procedures. Ten randomly selected radiographs and ten randomly selected clinical photographs were reassessed by the same examiner after a 10-day interval. Intra-examiner reliability was assessed using a two-way mixed-effects intraclass correlation coefficient for absolute agreement and single measurements. The ICC was 0.9989 (95% CI: 0.9959–0.9997) for MBL measurements and 0.9698 (95% CI: 0.8872–0.9924) for total PES scores.

### Statistical analysis

Marginal Bone Level (MBL) and Pink Esthetic Score (PES) were analyzed using repeated measure analysis of variance (RM-ANOVA). Marginal bone level and pink esthetic scores were reported as mean ± standard deviation. To handle missing data, a random imputation 5-cycle technique was used. If RM-ANOVA was significant, pairwise comparisons were performed and adjusted utilizing Bonferroni corrections. The statistical significance level was set at *p* ≤ 0.05. Statistical Analysis was performed using SPSS 29 (IBM Corp.)

## Results

This retrospective study included a total of 108 patients. The analyzed sample underwent the placement and monitoring of a total of 193 implants with a convergent transmucosal profile. Gender distribution analysis showed that 66 implants were placed in male patients and 127 in female patients. Participant age at the time of surgery ranged from a minimum of 31 to a maximum of 88 years, with a mean age of 61 ± 13 years. It should be specified that demographic age calculations were related to the time of placement of each implant; therefore, in patients who underwent multiple surgical interventions at different time points (delayed placements), the same individual contributed to the calculation with the specific age corresponding to each surgical session, thus reflecting the patient’s biological condition at the time of each intervention.

At short-, medium-, and long-term follow-up, MBL measurements were available for 124 implants in 80 patients, 92 implants in 58 patients, and 32 implants in 23 patients, respectively. PES assessments were available for 132 implants in 85 patients at baseline, 111 implants in 68 patients at short-term, 59 implants in 42 patients at medium-term, and 23 implants in 16 patients at long-term follow-up.

Regarding intraoral topographic distribution, the majority of implants (164) were placed in posterior regions, whereas 28 implants were inserted in anterior regions.

Analysis of the raw data revealed variability in the sample size available for radiographic (MBL) and esthetic (PES) evaluations over the observation period, due to missing data or drop-out at intermediate and long-term follow-up visits.

**Fig. 3:**
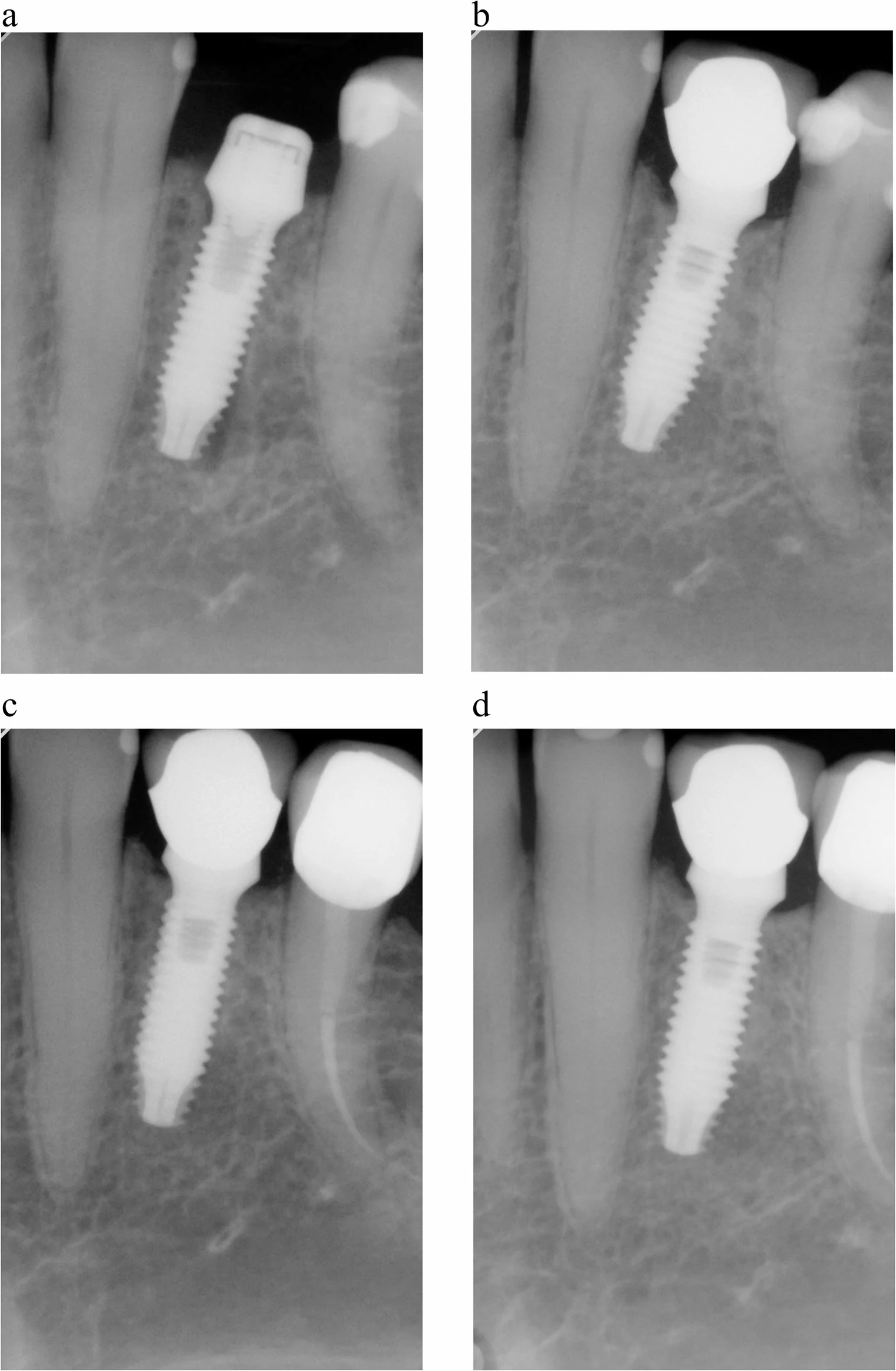
Periapical radiographic images of the same implant at baseline (implant placement) (**a**), short term (**b**), medium term (**c**), and long term (**d**)

**Fig. 4:**
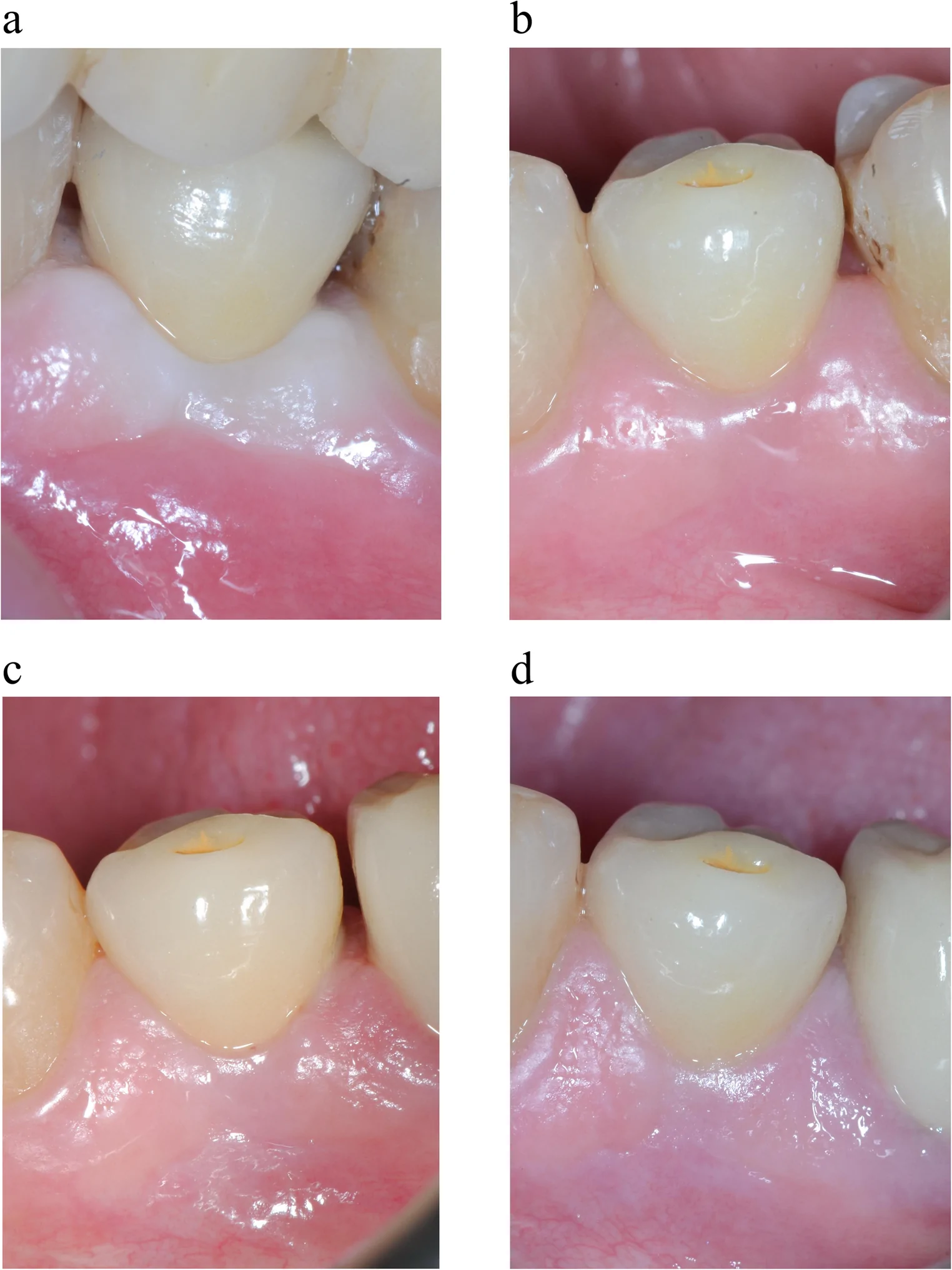
Buccal photographs of the same implant obtained at baseline (prosthetic loading) (**a**), short-term (**b**), medium-term (**c**), and long-term (**d**)

- **Marginal Bone Level (MBL)**: Out of the total number of implants placed, valid data at short-term follow-up (MBL Short) were available for 124 implants. At medium-term follow-up (MBL Medium), the analyzable sample was reduced to 92 implants, while at long-term follow-up (MBL Long), 32 implants were analyzed. (Figs. 3a, b,c, d)
- **Pink Esthetic Score (PES)**: Esthetic evaluation was performed on a subset of implants for which complete photographic documentation was available. At baseline (PES Baseline), 132 implants were assessed. Subsequent follow-up evaluations included 111 implants at short-term follow-up (PES Short), 59 at medium-term follow-up (PES Medium), and 23 at long-term follow-up (PES Long). (Figs. 4a, b,c, d)

### Marginal bone level (MBL)

Descriptive statistics for MBL at different time points showed relatively stable means and standard deviations (Table 1).

**Table 1:** Descriptive Statistics of Marginal Bone Level (MBL) at the Different Time Points

Time point	Mean (mm)	Standard Deviation (mm)
Short term (1–2 years)	1.1725	0.83136
Medium term (3–6 years)	1.1677	1.05492
Long term (7–10 years)	1.226	0.87965

The multivariate test on the effect of MBL over time revealed no statistically significant differences (F = 0.794, df = 2.000, Sig. = 0.454). Mauchly’s Test of Sphericity indicated a violation of the sphericity assumption (W = 0.907, Chi-Square = 18.583, df = 2, Sig. < 0.001). However, within-subjects effects tests, even after Greenhouse-Geisser (Sig. = 0.484) and Huynh-Feldt (Sig. = 0.486) corrections, confirmed that there was no statistically significant change in MBL over time (Sphericity Assumed: F = 0.702, Sig. = 0.496).

Pairwise comparisons between time points further supported these findings (Table 2), showing non-significant differences in all comparisons (e.g., MBLS vs. MBLM: Mean Difference = 0.005, Sig. = 1.000; MBLS vs. MBLL: Mean Difference = −0.054, Sig. = 0.737).

**Table 2:** Pairwise Comparisons of Marginal Bone Level (MBL) Across Different Time Points (Bonferroni Correction)

**Comparison**	**Mean Difference**	**Standard Error**	**Significance (Sig.)**
MBLS vs MBLM	0.005	0.057	1.000
MBLS vs MBLL	−0.054	0.046	0.737
MBLM vs MBLL	−0.058	0.060	1.000

MBLS=MBL short term; MBLM=MBL medium term; MBLL=MBL long term

These data indicate that marginal bone level around the implants remained stable throughout the 10-year follow-up period.

###  Pink esthetic score (PES)

Descriptive statistics for PES at different time points showed an increasing trend (Table 3).

**Table 3:** Descriptive Statistics of the Pink Esthetic Score (PES) at the Different Time Points

Time point	Mean (mm)	Standard Deviation (mm)
Baseline (prosthetic load)	7.9932	2.58217
Short term (1–2 years)	9.7883	2.35895
Medium term (3–6 years)	10.2698	2.33992
Long term (7–10 years)	10.3448	2.39223

The multivariate test on the effect of PES over time showed an overall statistically significant difference (F = 61.202, df = 3.000, Sig. = <0.001). As with MBL, Mauchly’s Test of Sphericity revealed a violation of the sphericity assumption (W = 0.334, Chi-Square = 208.923, df = 5, Sig. < 0.001). However, within-subjects effects tests, even after corrections (Greenhouse-Geisser: F = 98.028, Sig. = <0.001; Huynh-Feldt: F = 98.028, Sig. = <0.001), confirmed a statistically significant change in PES over time (Sphericity Assumed: F = 98.028, Sig. = <0.001). Within-subjects contrasts tests revealed a significant linear effect (F = 145.739, Sig. = <0.001), indicating an increasing trend in PES.

Pairwise comparisons highlighted a statistically significant increase in PES from baseline at all subsequent time points considered. A further significant improvement in esthetic parameters was recorded when transitioning from the short-term assessment to the medium- and long-term evaluations. However, the comparison between medium-term and long-term follow-up did not reveal statistically significant differences, suggesting the achievement of stability in esthetic results after the maturation phase (Table 4).

**Table 4:** Pairwise Comparisons of the Pink Esthetic Score (PES) Across Different Time Points

Comparison	Mean Difference	Standard Error	Significance (Sig.)
PESB vs. PESS	−1.795*	0.163	< 0.001
PESB vs. PESM	−2.277*	0.174	< 0.001
PESB vs. PESL	−2.352*	0.198	< 0.001
PESS vs. PESM	−0.482*	0.072	< 0.001
PESS vs. PESL	−0.557*	0.164	0.005
PESM vs. PESL	−0.075	0.140	1.000

PESB = PES baseline; PESS = PES short term; PESM = PES medium term; PESL = PES long term

These results demonstrate a significant improvement in soft tissue aesthetics (PES) that consolidates in the first few years after prosthetic loading and remains stable in the long term.

## Discussion

This long-term (10-year) retrospective study aimed to evaluate the stability of hard and soft tissues around implants featuring a convergent transmucosal neck design. The main findings indicate not only excellent marginal bone level (MBL) stability over the decade, but also a significant improvement in esthetic parameters (PES) over time, suggesting a positive adaptive response of peri-implant tissues to this specific macro-geometry.

The marginal bone stability observed in the present study is consistent with the biological concepts underlying platform switching (PS). It is well documented that the horizontal displacement of the implant–abutment interface toward the central axis of the implant moves the inflammatory infiltrate and the microgap away from the crestal bone, thereby preserving marginal bone levels [16]. The convergent implant neck design analyzed in this study acts as an intrinsic, amplified form of PS, creating a physical space for both hard and soft tissues. This finding is in line with a recent systematic review reporting that convergent or concave transmucosal profiles are associated with significantly less bone loss compared with divergent or traditional parallel profiles.Valente et al., [33] Moreover, comparative clinical studies have shown that implants with a convergent collar exhibit reduced peri-implant bone loss compared with those with a divergent collar, suggesting that the morphology of the transmucosal area plays a crucial role in bone preservation [2].

A relevant outcome of this study is the improvement of the Pink Esthetic Score (PES) over time. This phenomenon may be explained by the ability of the convergent profile to create a “chamber” that allows growth and thickening of peri-implant soft tissues. The literature suggests that concave or convergent transmucosal components promote soft tissue stability [28] and favor the formation of a circular collagen fiber seal (“O-ring effect”) that protects the underlying bone [9]. In addition, it has been demonstrated that the use of concave or customized emergence profiles, even at early stages such as healing abutments, leads to superior outcomes in terms of papilla index and soft tissue stability compared with standard divergent profiles.Perez et al., [24] This concept is further supported by evidence indicating that increased soft tissue thickness represents a protective factor for the crestal bone(Linkevicius et al., 2009; [25]); by virtually increasing the space available for the mucosa, the convergent design may compensate for thin biotypes, contributing to the stability observed.

The convergent neck design also offers significant advantages from a prosthetic perspective, allowing clinicians to modulate the crown emergence profile. In contrast, convex or excessively divergent emergence profiles have been associated with an increased risk of mucosal recession [31] and greater marginal bone loss [32]. Furthermore, wide emergence angles have been correlated with a higher incidence of peri-implant mucositis [29]. The convergent geometry of the implant used in this study facilitates a tissue-respecting prosthetic approach, allowing the crown to emerge with a gradual profile that supports the soft tissues without exerting excessive pressure.

The implant neck morphology analyzed in this study allows for an approach comparable to vertical preparation techniques on natural teeth like Biologically Oriented Preparation Technique (B.O.P.T.), in which soft tissues adapt to the prosthetic shape rather than to a predetermined finish line [18]. This characteristic enables flexible prosthetic management, which is useful not only for immediate esthetic outcomes but also for the management of long-term complications. Recent studies have highlighted that residual craniofacial growth and continuous dental eruption may lead to vertical discrepancies (infraocclusion) between implants and natural teeth even in adult patients [34]. In this context, tissue-level implants with a convergent transmucosal profile may offer additional prosthetic flexibility. When replacement of an infraoccluded crown is required, the margin of the new restoration can be positioned more coronally along the convergent transmucosal profile than that of the previous restoration, potentially allowing adaptation to the new clinical conditions without the need for deep submersion of the restorative margin or additional surgical procedures [34].

Although the 10-year clinical outcomes are encouraging, this study presents several inherent limitations that must be discussed to allow a correct interpretation of the data.

The main limitation lies in the retrospective nature of the analysis. In addition, the absence of a contemporary control group (e.g., implants with flat-to-flat connections or divergent neck profiles placed in the same patients or under comparable conditions) does not allow us to state with absolute certainty that the favorable outcomes observed are exclusively attributable to the convergent macro-geometry. Bone stability was assessed using periapical radiographs. Although this method is widely accepted for monitoring interproximal (mesial and distal) bone levels, it does not provide information on the buccal and lingual bone walls. Clinical studies employing cone beam computed tomography (CBCT) have demonstrated that the dimensions of the facial bone crest are critical for esthetic outcomes and may vary independently of interproximal bone peaks. It should also be acknowledged that the cervical profile of the implant is not uniformly convergent along its entire transmucosal portion. In particular, especially in implants with narrower diameters, a very limited initial divergent segment is present before the profile becomes convergent. Therefore, the geometry evaluated in this study should be interpreted as predominantly, rather than completely, convergent.

A significant confounding factor in a long-term study is the variability in prosthetic design. Recent evidence indicates that the prosthetic emergence angle and crown contour (convex vs. concave) play a crucial role in peri-implant health and tissue stability. As the emergence angle of each prosthetic restoration was neither standardized nor specifically measured over the 10-year period, it was not possible to quantify the extent to which the specific crown design contributed to clinical success or to potential complications, independently of the implant neck geometry. In addition, the vertical position of the prosthetic margin along the transmucosal implant profile was not standardized or quantitatively recorded. Consequently, the potential influence of the effective depth of the resulting transmucosal concavity and of the individual crown–implant relationship on MBL and PES outcomes could not be specifically assessed.

The literature has extensively documented that baseline mucosal thickness (biotype) is a key determinant of initial crestal bone remodeling; thin soft tissues (< 2 mm) are often associated with greater bone loss due to the establishment of the biological width. In this retrospective study, it was not possible to retrieve accurate data on soft tissue thickness at the time of implant placement for all patients. Consequently, it cannot be determined whether the observed stability was predominantly influenced by the convergent implant geometry or by the presence of a thick biotype in a substantial proportion of the treated patients.

Finally, although a 10-year follow-up is substantial, the final number of implants analyzed may not be sufficient to detect rare events or late complications with statistical significance. Moreover, as in many long-term studies, the influence of uncontrolled variables occurring over the decade (such as changes in oral hygiene habits or the onset of minor systemic conditions) on tissue stability cannot be excluded.

Despite the limitations described, the present study provides a significant contribution to the implant dentistry literature. Although the biological advantages of convergent or concave profiles—such as reduced marginal bone loss and improved soft tissue stability—have been documented in short- and medium-term studies [28], longitudinal data confirming the long-term stability of these outcomes are largely lacking. To the best of our knowledge, this is the first study to document the clinical behavior of tissue-level implants with a convergent transmucosal profile over a time span exceeding 10 years. The confirmation that marginal bone stability and improvements in esthetic parameters are not transient, but are maintained or even enhanced over a decade, biologically validates the concept of transmucosal convergence as a reliable strategy for the long-term preservation of peri-implant health.

## Conclusions

Within the limits of the present retrospective study, the results observed over a 10-year follow-up indicate that the use of *tissue level* implants with a convergent transmucosal profile is associated with marginal bone level stability and a progressive improvement in soft tissue esthetic parameters.

These clinical observations suggest that the specific convergent macro-geometry of the implant neck may favor a positive adaptation of peri-implant tissues, likely creating adequate space for mucosal thickening and consequent protection of the underlying crestal bone. Whether this implant configuration may provide particular benefits in sites with a thin peri-implant mucosal phenotype remains an interesting hypothesis that should be specifically investigated in prospective controlled studies.

However, further prospective randomized clinical trials are needed to confirm these results and to validate them on a larger scale.

## Data Availability

No datasets were generated or analysed during the current study.
